# Increased frequency of IL-6-producing non-classical monocytes in neuromyelitis optica spectrum disorder

**DOI:** 10.1186/s12974-017-0961-z

**Published:** 2017-09-25

**Authors:** Byung Soo Kong, Yeseul Kim, Ga Young Kim, Jae-Won Hyun, Su-Hyun Kim, Aeran Jeong, Ho Jin Kim

**Affiliations:** 1 Division of Clinical Research, Research Institute and Hospital of the National Cancer Center, 323 Ilsan-ro, Ilsandong-gu, Goyang, 10408 South Korea; 2 Department of Neurology, Research Institute and Hospital of the National Cancer Center, Goyang, South Korea

**Keywords:** Neuromyelitis optica spectrum disorder, Monocyte, Non-classical, IL-6, Aquaporin-4

## Abstract

**Background:**

Neuromyelitis optica spectrum disorder (NMOSD) is an autoimmune inflammatory disease of the central nervous system that preferentially affects the optic nerves, spinal cord, and area postrema. A series of evidence suggested that B cells play a fundamental role in the pathogenesis of NMOSD. However, there are still gaps left to be answered in NMOSD pathogenesis suggesting the roles of other immune cells. This study aimed to investigate the monocyte inflammatory characteristics, monocyte subset frequency and cytokine production, and cell-surface molecule expression in NMOSD, multiple sclerosis (MS), and healthy controls (HC).

**Methods:**

Peripheral blood mononuclear cells of 20 aquaporin 4IgG-positive NMOSD patients, 20 MS patients, and 20 healthy controls were collected to analyze the monocyte subsets and to purify monocytes. To mimic the adaptive immunity, we have activated the monocytes using CD40L and IFN-γ to observe the production of cytokines and expression of cell-surface molecules.

**Results:**

NMOSD monocytes showed a remarkable increase in the production of pro-inflammatory cytokines (IL-6, IL-1β) and increased expression of cell-surface molecules (CD80, HLA, ICAM-1, CD16), as well as a decrease in the levels of anti-inflammatory cytokine IL-10, compared to healthy control (HC) monocytes. As expected, MS monocytes also exhibit increased inflammatory cytokine production and increased cell-surface molecule expression compared to HC monocytes. Further analysis of monocyte subsets revealed that NMOSD monocytes have an increased frequency of the non-classical monocyte subset (CD14^+^CD16^++^) and a decreased frequency of the classical monocyte subset (CD14^++^CD16^+^) compared to HC monocytes. This finding was distinctly different from that of MS monocytes, which had an increased intermediate monocyte (CD14^+^CD16^+^) subset. In addition, these NMOSD non-classical monocyte subsets were highly dedicated, IL-6-producing monocytes.

**Conclusions:**

Increased expression of cell-surface molecules and a reciprocal dysregulation of inflammatory and anti-inflammatory cytokines in NMOSD monocytes suggest an altered monocyte inflammatory response. CD14^+^CD16^++^ non-classical monocyte subset was more abundant in NMOSD monocytes than in HC or MS monocytes, and NMOSD non-classical monocyte subset had dysregulated IL-6 production, a phenotype which has been reported to be highly associated with NMOSD pathogenesis.

**Electronic supplementary material:**

The online version of this article (10.1186/s12974-017-0961-z) contains supplementary material, which is available to authorized users.

## Background

Neuromyelitis optica spectrum disorder (NMOSD) is an autoimmune inflammatory disease of the central nervous system (CNS) characterized by the generation of autoantibodies against aquaporin-4 (AQP4), which is the most abundant water channel in the CNS [1], severe immune-mediated demyelination, and axonal damage that preferentially targets the optic nerves and spinal cord [2]. Previously regarded as a variant of multiple sclerosis (MS) [3], NMOSD is now considered an independent disease entity [2]. Binding of AQP4-IgG or NMO-IgG, which are predominantly IgG_1_, to the astrocytic AQP4, causes complement-dependent cytotoxicity and secondary inflammation, with granulocyte and macrophage infiltration, blood-brain barrier disruption, and oligodendrocyte injury [2].

NMOSD has been frequently associated with dysregulated production of cytokines [4], which are produced from numerous immune cells, including monocytes [5]. Among these cytokines, IL-6 is presumed to be critical in the pathogenesis of NMOSD because it is significantly elevated in the serum and cerebrospinal fluid (CSF) of NMOSD patients and promotes AQP4-IgG production by plasmablasts [6], and blockade of IL-6 in NMOSD patients has been reported to be effective in preventing disease relapse [6]. One of the proposed mechanisms of IL-6 in disease pathogenesis is that it regulates the transition of leukocyte recruitment. IL-6 shifts neutrophilic infiltrate to the mononuclear cell infiltrate and leads them to participate in disease pathogenesis [7].

Monocytes are myeloid cells that have multiple immunological functions, including antigen presentation, phagocytosis, and cytokine production. Recent reports suggest that upon activation with LPS, NMOSD monocytes produce higher amounts of inflammatory cytokines such as IL-12, IL-23, and IL-6 compared to healthy controls (HC) [8]. Also, NMOSD monocytes exhibited increased expression of certain co-stimulatory molecules compared to healthy controls [9]. However, these data did not show how monocytes react in adaptive immunity, which could be more relevant to study NMOSD pathogenesis where monocytes are recruited to the inflammation site by IFN-γ and activated through CD40/CD40L ligation [10, 11]. Monocytes possess receptors for the IgG Fc fragment that regulate IgG antibodies in autoimmune diseases [12]. Among these receptors, CD16, or FcγRIII, is highly expressed in monocytes and used to determine monocyte subset populations and has a higher affinity for IgG_1_ than other IgG subtypes [13]. Monocytes are classified into three subsets based on their CD14 and CD16 expression: classical (CD14^++^CD16^+^), intermediate (CD14^+^CD16^+^), and non-classical (CD14^+^CD16^++^). This classification of monocytes is relatively recent and the individual roles and function of each subset are largely unknown, but CD16 has been linked to susceptibility to autoimmune diseases [14, 15]. Also, it has been recently published that in Chinese population, non-classical monocyte frequency was higher than HC [16]. CD16^+^ monocytes have been reported to facilitate T cell migration and found around blood vessels in active MS lesions [17]. Taken together, these data suggest that CD16^+^ monocytes could also play an important role in NMOSD.

In this study, we investigated the monocyte inflammatory characteristics, monocyte subset frequency and cytokine production, and cell-surface molecule expression in NMOSD, MS, and healthy controls. We found a remarkable increase in the levels of pro-inflammatory cytokines (TNFα, IL-6, IL-1β, IL-23) and a reciprocal decrease in the levels of an anti-inflammatory cytokine (IL-10) in NMOSD monocytes compared to HC monocytes by IFN-γ or CD40L activation. In addition, increased expression of CD80, ICAM-1, and HLA-DR occurred upon activation with IFN-γ or CD40L, and a constitutively high expression level of CD16 was observed in NMOSD monocytes compared to HC monocytes. Further analysis of monocyte subsets revealed that the high expression of CD16 in NMOSD monocytes, in fact, resulted from an increased frequency of non-classical monocytes. These non-classical monocytes were discovered to be constitutively IL-6-producing cells. Our findings indicate that NMOSD monocytes have increased inflammatory cytokine production, increased inflammatory cell-surface molecule expression, and an increased frequency of a non-classical monocyte subset.

## Methods

### Patients and healthy controls

Twenty patients with NMOSD fulfilling 2015 diagnostic criteria for NMOSD [18] and 20 patients with relapsing-remitting MS fulfilling the revised McDonald criteria [19] were enrolled from the Department of Neurology, National Cancer Center, South Korea. None of the patients had received high-dose steroids within 2 months preceding blood draws. Twenty age- and sex-matched healthy controls were also recruited for blood donation. Demographic and clinical characteristics of three groups and basic information of each patient are summarized in Tables 1 and 2, respectively. Peripheral blood was obtained by venipuncture and processed immediately for monocyte purification as described below.

**Table 1:** Baseline and clinical characteristics of the study population

All participants (*n* = 60)						
Disease category	No. of subjects	Age (years, mean ± SD)	F:M (*n*)	EDSS (median)	Disease duration (mean ± SD)	AQP4 positivity (%)
HC	20	33.9 ± 10.7 (23–48)	16:4	n/a	n/a	n/a
MS	20	33.4 ± 8.2 (19–51)	16:4	2.5	60.4 ± 42.45	0%
NMOSD	20	36.4 ± 6.2 (28–50)	16:4	2.5	102.1 ± 84.10	100%

**Table 2:** Information on immunosuppressive treatments, sampling date, last relapse date, relapse status, and EDSS score of NMOSD and MS patients

Patient number	Disease	Age	Sex	Treatment	Days passed from onset to sampling date (d)	Disease duration (m)	Relapse or stable	EDSS score
1	NMOSD	35	F	MMF	60	90	Stable	2
2	NMOSD	36	F	MFF	850	72	Stable	1.5
3	NMOSD	41	F	PD	197	6	Stable	3.5
4	NMOSD	33	M	MMF	1767	216	Stable	4
5	NMOSD	30	F	MMF	565	139	Stable	1.5
6	NMOSD	39	M	MMF	203	111	Stable	7.5
7	NMOSD	44	F	AZA	63	8	Stable	0
8	NMOSD	28	F	RTX	282	130	Stable	1
9	NMOSD	36	F	PD	79	7	Stable	2
10	NMOSD	30	F	MMF	834	64	Stable	2
11	NMOSD	50	F	AZA	418	13	Stable	5
12	NMOSD	36	F	MMF	1783	266	Stable	2
13	NMOSD	46	M	RTX	69	15	Stable	3
14	NMOSD	32	F	AZA	475	113	Stable	2
15	NMOSD	40	F	MMF	799	235	Stable	3
16	NMOSD	34	F	PD	60	7	Stable	2.5
17	NMOSD	31	F	MMF	817	49	Stable	2
18	NMOSD	29	F	MMF	1403	50	Stable	1.5
19	NMOSD	46	F	RTX	1753	206	Stable	2
20	NMOSD	33	M	RTX	64	245	Stable	2.5
21	MS	42	F	Glatiramer acetate	71	78	Stable	3.5
22	MS	38	F	Teriflunomide	87	84	Stable	4
23	MS	30	M	Naïve	113	77	Stable	3
24	MS	27	F	Interferon-β	904	85	Stable	5
25	MS	35	F	Interferon-β	2014	161	Stable	4
26	MS	19	M	Glatiramer acetate	208	29	Stable	2
27	MS	25	M	Naïve	60	3	Stable	2
28	MS	23	F	Interferon-β	405	13	Stable	3.5
29	MS	23	F	Glatiramer acetate	424	13	Stable	2
30	MS	46	F	Interferon-β	1888	76	Stable	0
31	MS	33	F	Naïve	60	31	Stable	2.5
32	MS	31	F	Interferon-β	284	18	Stable	2.5
33	MS	51	F	Interferon-β	872	121	Stable	2.5
34	MS	34	F	Interferon-β	739	35	Stable	0
35	MS	34	M	Interferon-β	225	25	Stable	2
36	MS	26	F	Interferon-β	206	40	Stable	0
37	MS	44	F	Interferon-β	191	10	Stable	0
38	MS	36	F	Interferon-β	1508	151	Stable	1.5
39	MS	36	F	Interferon-β	519	43	Stable	3.5
40	MS	34	F	Interferon-β	840	115	Stable	2

### PBMC and monocyte isolation and culture

Human peripheral blood mononuclear cells (PBMCs) were obtained using a Ficoll density gradient (GE Healthcare, Pasching, Austria) of buffy coats from healthy donors, NMOSD, and MS patients. Monocytes were purified using a CD14^+^ magnetic separation system (MACS, Miltenyi Biotec, Sunnyvale, CA, USA). The purity and viability of purified cells were assessed by flow cytometry and PI staining, respectively. All monocyte and PBMC cultures were performed in RPMI-1640 supplemented with 10% heat-inactivated fetal bovine serum (HyClone Laboratories, Logan, UT, USA) and 1% penicillin/streptomycin (GE Healthcare, Pasching, Austria).

For cytokine production and surface molecule analysis, CD14^+^ purified monocytes (5.0 × 10^5^ cells/mL) were incubated for 24 h at 37 °C in complete medium. Monocytes were left unstimulated or were stimulated with recombinant human IFN-γ (100 ng/mL, R&D systems, Minneapolis, MN, USA), or recombinant human CD40L (1.0 μg/mL, Enzo Life Sciences, Farmingdale, NY, USA) or both, to mimic encounters with activated T cells. Supernatants were collected for cytokine detection, and cells were detached for flow cytometry analysis. For ICS, CD14^+^ purified monocytes (5 × 10^6^ cells/mL) were cultured in complete medium, with or without CD40L, for 6 h.

### Cytokine detection in monocyte cultured media

The supernatant from CD14^+^ monocytes cultured media were collected and stored at − 80 °C before use. Cytokine measurement was performed within 4 weeks after the collection of monocytes cultured media and aliquoted to appropriate amounts to avoid repeated freeze/thaw cycles. For measurement of cytokines, standard ELISA kits for IL-10, IL-6, IL-1β, TNFα (all from BioLegend, San Diego, CA, USA), and IL-23 (eBioscience, Vienna, Austria) were purchased and used according to the manufacturer’s instructions.

### Flow cytometry assays

Purified CD14^+^ monocytes cultured for 24 h, with or without stimulation, were labeled with primary antibodies directed against human CD80, CD86, HLA-DR, ICAM-1, or CD16 (all from BD Biosciences, San Jose, CA, USA). For ex vivo staining of inflammatory surface molecules, PBMCs were stained with CD3, CD14, CD16, CD56, CD66b, CD80, CD86, HLA-DR, and ICAM-1. For gating of the three monocyte subsets, we used a fixed set of CD14, CD16, CD3, CD19, CD56, and CD66b (all from BD Biosciences) on PBMCs for negative selection. For intracellular cytokine staining (ICS), PBMCs and monocytes were isolated using a MACS pan-monocyte isolation kit (Miltenyi Biotec, Bergisch Gladbach, Germany) and were incubated with Bredfeldin A (GolgiPlug; 1 μg/mL; BD Biosciences) and CD40L for 6 h. After being stained for surface markers, cells were fixed and made permeable according to the manufacturer’s instructions (BD Biosciences). IL-6, IL-10, TNFα, and IL-1β antibodies against cytokines were from BD Biosciences and IL-23 from Biolegend (San Diego, CA, USA). The appropriate Ig isotypes were included as negative controls. Data was acquired using a FACSVerse (BD Biosciences) and were analyzed with Flowjo software (Treestar, Ashland, OR, USA).

### SPADE analysis

Spanning-tree Progression Analysis of Density-normalized Events (SPADE) is an automated clustering algorithm to confirm manual gating [20]. SPADE analyzes raw flow cytometry data to agglomerate different types of surface molecules into clusters, which allows for easy visualization of rare events. To implement this analysis, we fixed the default settings of 100 nodes and 5 × 10^4^ cells per sample.

### AQP4-IgG assay

The AQP4-IgG serostatus was confirmed by an in-house cell-based assay using the M23-AQP4-transfected Human Embryonic Kidney 293 (HEK-293) cell line, generated by an internal ribosome entry site (IRES) vector, as previously reported [21].

### Statistical analysis

Data are presented as mean ± standard error of mean (SEM). Prism software was used for unpaired one-way or two-way analysis of variance to assess significance between groups, and Dunnett’s multiple comparison was performed. A *p* value of less than 0.05 was considered statistically significant and designated as follows: *p < 0.05, **p < 0.01, ***p < 0.001, and ****p < 0.0001.

## Results

### Increased inflammatory cytokine production and decreased IL-10 production in NMOSD monocytes

**Fig. 1:**
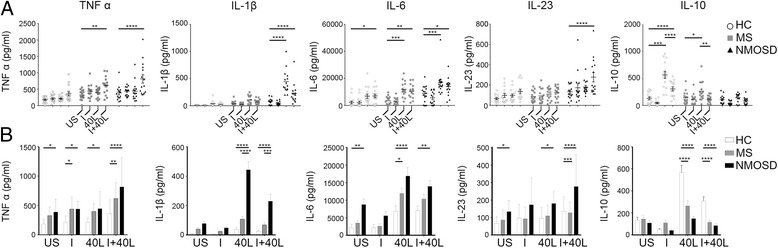
Cytokine production in healthy control (HC), MS, and NMOSD patient monocytes. Monocytes were purified using MACS CD14^+^ isolation kit from PBMCs. Purified CD14^+^ monocytes were incubated, with or without IFN-γ or CD40L stimulation for 24 h. Supernatants were obtained to analyze the production of five different cytokines (TNFα, IL-1β, IL-6, IL-23, and IL-10; pg/mL) (**a**) and show a comparison in cytokine production between three groups (*n* = 15) (**b**). Graphs were created using Flowjo and Prism software. Assessment of statistical significance was performed by two-way ANOVA. Data are mean ± S.E.M of 15 subjects each. *p < 0.05, **p < 0.01, ***p < 0.001, ****p < 0.0001. US unstimulated, I interferon-γ, 40 L CD40L

To evaluate the reactivity of monocytes to inflammatory stimuli, monocytes isolated from PBMCs of healthy control (HC), as well as MS and NMOSD patients (Additional file 1: Figure S1-A), were stimulated with IFN-γ and/or CD40L (*n* = 15 each) to mimic the inflammatory state, as previously reported [22, 23]. In the inflammatory state, monocytes are recruited to the site of inflammation by IFN-γ and activated through CD40/CD40L ligation by other immune cells such as B and T cells [10, 11]. The data of cytokine ELISA showed that IFN-γ stimulation by itself did not induce an appreciable amount of inflammatory cytokine production from monocytes, whereas it inhibited the CD40L-induced production of IL-10 in HC and MS (*p* < 0.01) (Fig. 1a). Unlike IFN-γ, CD40L stimulation by itself increased the production of IL-1β (*p* < 0.0001) and IL-6 (*p* < 0.0001) from NMOSD monocytes compared to unstimulated (US) monocytes. Production of IL-6 was also greatly increased following CD40L stimulation compared to that in US in MS monocytes (*p* < 0.001), whereas only a modest increase was seen in IL-10 production in MS. There was no increase in IL-10 production for NMOSD monocytes. Co-stimulation with IFN-γ and CD40L showed different reactivity and diversity of pro-inflammatory cytokine production (Fig. 1a). In NMOSD monocytes, TNFα (*p* < 0.0001), IL-1β (*p* < 0.0001), IL-6 (*p* < 0.05), and IL-23 (*p* < 0.0001) levels were all increased by co-stimulation compared to that in US. In MS monocytes, TNFα (*p* < 0.01) and IL-6 (*p* < 0.01) levels were increased by co-stimulation compared to that in US (Fig. 1a). In HC monocytes, only a modest increase in IL-6 production was observed after co-stimulation (*p* < 0.05). In contrast, a marked induction of IL-10 production by CD40L stimulation was observed (*p* < 0.0001). Overall, NMOSD monocytes exhibit a greater inflammatory response to IFN-γ and/or CD40L stimulation than HC and MS monocytes, also showing an increased production of pro-inflammatory cytokines, and an impaired induction of IL-10 following CD40L stimulation, all of which suggest the existence of dysregulated cytokine production in NMOSD monocytes.

To confirm the dysregulated cytokine production in NMOSD monocytes, we then compared cytokine production between HC, MS, and NMOSD monocytes. Even in the US condition, NMOSD monocytes produced a significantly higher amount of TNFα (*p* < 0.05), IL-6 (*p* < 0.01), and IL-23 (*p* < 0.05) than HC monocytes (Fig. 1b). Following IFN-γ stimulation, TNFα production was significantly higher in MS monocytes than in HC monocytes (*p* < 0.05), as previously reported [5]. Likewise, NMOSD monocytes also showed increased production of TNFα compared to HC monocytes (*p* < 0.05). Following CD40L stimulation, NMOSD monocytes produced a significantly higher amount of all pro-inflammatory cytokines, most markedly IL-1β (*p* < 0.0001) and IL-6 (*p* < 0.0001), and a markedly lower amount of IL-10 (*p* < 0.0001) compared to HC monocytes. In MS, only IL-6 production was higher than HC monocytes after CD40L stimulation (*p* < 0.05). This result corresponds to the previous reports on IL-6 and MS, which showed IL-6 production was similar between MS and HC monocytes in US, but monocyte activation led to a significant increase in IL-6 levels in MS [24]. In addition, after CD40L stimulation, IL-1β production was far greater (*p* < 0.0001) in NMOSD monocytes than in MS monocytes (Fig. 1b). Co-stimulation with IFN-γ and CD40L in NMOSD also showed a significant difference in cytokine production (higher in TNFα, *p* < 0.0001; IL-1β, *p* < 0.0001; IL-6, *p* < 0.01; and IL-23 *p* < 0.0001, and lower in IL-10, *p* < 0.0001) compared to that in HC monocytes, but there was no synergistic effect on cytokine production. Co-stimulation resulted in decreases in IL-10 production, compared to CD40L stimulation of HC (*p* < 0.0001) and MS (*p* < 0.01) monocytes alone, which corresponds to previous findings that IFN-γ inhibits IL-10 production (Fig. 1a) [25].

**Fig. 2:**
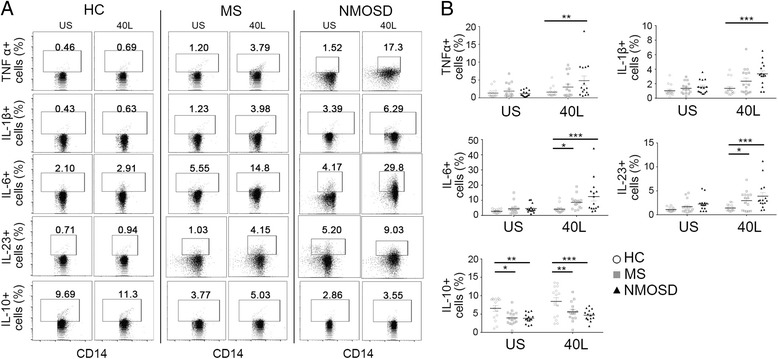
Intracellular staining of healthy control (HC), MS, and NMOSD patient monocytes. Monocytes purified with MACS pan-monocyte isolation kit were treated with or without CD40L (1.0 μg/mL) for 6 h and stained with antibodies to the appropriate surface antigen and anti-cytokine antibodies (TNFα, IL-1β, IL-6, IL-23, IL-10). The graphs shown are representative graphs (**a**) and show a comparison between three groups (*n* = 15) (**b**). Graphs were created using Flowjo and Prism software. Assessment of statistical significance was performed by two-way ANOVA. Data are mean ± S.E.M of 15 subjects each. *p < 0.05, **p < 0.01, ***p < 0.001, ****p < 0.0001. US unstimulated, I interferon-γ, 40 L CD40L

To confirm the ELISA data, we conducted ICS on HC, MS, and NMOSD monocytes (*n* = 15 each), which were purified to obtain pan-monocyte population (Additional file 1: Figure S1-B). For this experiment, we elected to only use CD40L because CD40L stimulation was more effective than IFN-γ at inducing monocyte cytokine production, and co-stimulation was not synergistic. The data showed that in NMOSD, there was an increase in the frequencies of TNFα^+^ (*p* < 0.01), IL-1β^+^ (*p* < 0.001), IL-6^+^ (*p* < 0.001), and IL-23^+^ (*p* < 0.001) monocytes following CD40L stimulation compared to that in CD40L-stimulated HC monocytes (Fig. 2a, b). In addition, MS also showed increased frequencies of IL-6^+^ (*p* < 0.05) and IL-23^+^ (*p* < 0.05) cells following CD40L stimulation compared to CD40L-stimulated HC monocytes. On the other hand, there were fewer IL-10^+^ monocytes observed for NMOSD and MS under both US (MS, *p* < 0.05; NMOSD, *p* < 0.01) and CD40L-stimulated conditions (MS, *p* < 0.01; NMOSD, *p* < 0.001) (Fig. 2a). Overall, the ICS analysis of NMOSD, MS, and HC monocytes confirmed the ELISA results, showing the increased production of pro-inflammatory cytokines and impaired induction of IL-10 in NMOSD.

### Increased expression of inflammatory cell-surface molecules in NMOSD monocytes

**Fig. 3:**
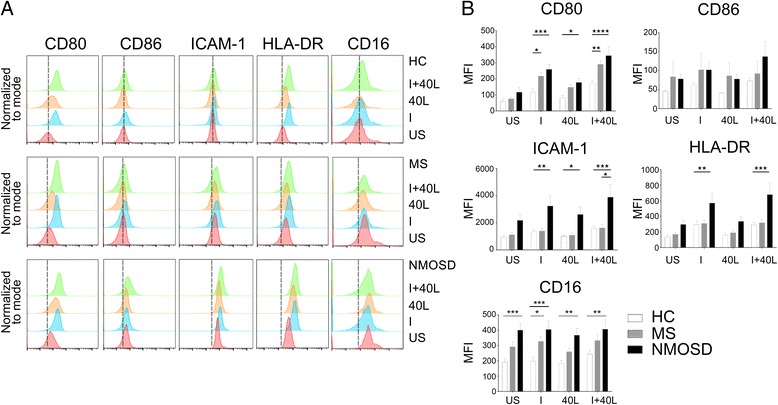
Differential expression of surface molecules on monocytes from healthy control (HC), MS, and NMOSD patients. Monocytes were purified using MACS CD14^+^ isolation kit from PBMCs. **a** Representative overlaid histograms of five different surface markers (CD80, CD86, ICAM-1, HLA-DR, CD16) after stimulation of HC, MS, or NMOSD monocytes with IFN-γ (100 ng/mL) and/or CD40L (1.0 μg/mL) for 24 h. The dashed lines represent the peak of fluorescence intensity in healthy US individuals. The histograms were created using Flowjo software. **b** Large-scale analysis of surface molecules comparing three groups (n = 15). Assessment of statistical significance was performed by two-way ANOVA. Data are mean ± S.E.M of 15 subjects each. *p < 0.05, **p < 0.01, ***p < 0.001, ****p < 0.0001. MFI median fluorescence intensity, US unstimulated, I interferon-γ, 40 L CD40L

Next, we compared the cell-surface expression levels of key immune molecules in CD14^+^ monocytes derived from HC, MS patients, and NMOSD patients (*n* = 15 each). In the US condition, NMOSD monocytes had expression levels of CD80, CD86, ICAM-1, and HLA-DR similar to that in HC and MS monocytes (Fig. 3a). However, IFN-γ stimulation of NMOSD monocytes increased the expression levels of CD80 (*p* < 0.001), ICAM-1 (*p* < 0.01), and HLA-DR (*p* < 0.01) compared to IFN-γ-stimulated HC monocytes. The level of CD80 in MS monocytes were also increased compared to that in HC monocytes (*p* < 0.05), as previously reported [26]. CD40L stimulation upregulated the expression of CD80 (*p* < 0.05) and ICAM-1 (*p* < 0.05) to higher levels in NMOSD monocytes than in HC monocytes. Similar to IFN-γ stimulation, co-stimulation with both CD40L and IFN-γ upregulated CD80 (*p* < 0.0001), ICAM-1 (*p* < 0.001), and HLA-DR (*p* < 0.001) expression in NMOSD monocytes compared to HC monocytes (Fig. 3b).

The level of CD16 was increased in NMOSD monocytes even in the US condition compared to HC monocytes (*p* < 0.001). However, neither CD40L nor IFN-γ stimulation could further alter the CD16 level; it remained constitutively high in NMOSD monocytes under all stimulated conditions when compared to stimulated HC monocytes. In contrast, MS monocytes only showed a difference in CD16 levels, compared to HC monocytes, following IFN-γ stimulation (*p* < 0.05) (Fig. 3a, b).

### Upregulated CD16 expression and increased frequency of non-classical monocytes (CD14^+^CD16^++^) in NMOSD-*

**Fig. 4:**
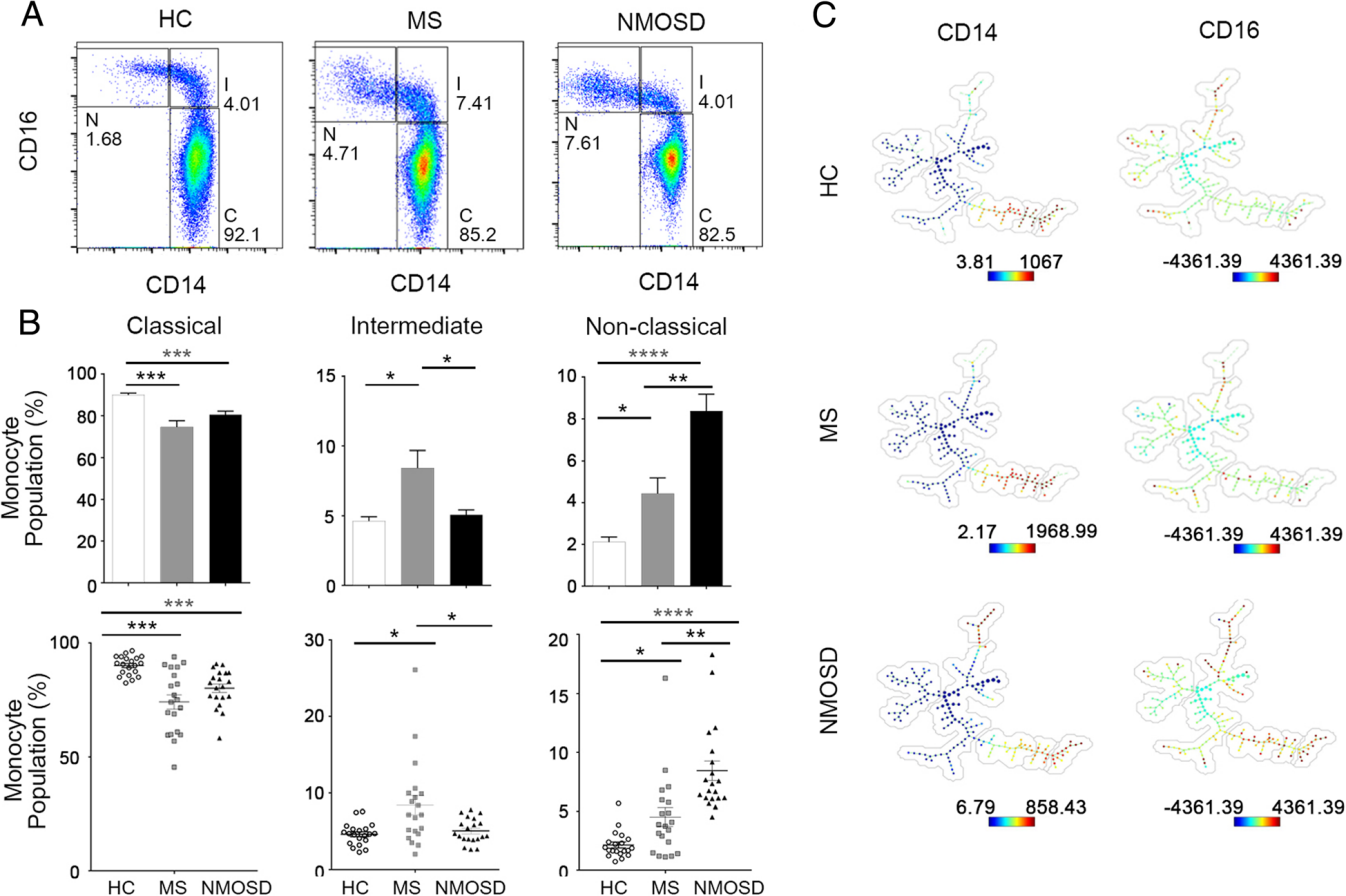
CD16 expression level of ex vivo monocytes. PBMCs were stained with cell-type markers (CD3, CD19, CD14, CD16, CD56, CD66b) to analyze the monocyte subset population of each group. Results are shown in **a** as representative graphs and **b** as a large-scale analysis to compare three groups (*n* = 20). Confirmation of manual gating was performed using the SPADE 3.0 program. The bubble around the nodes represents a particular cell type that was selected according to expression of its lineage marker, as indicated by the color code (with expression decreasing from red to blue) (**c**). NMOSD shows particular increase in CD16 expression compared to MS and HC. Graphs were created using the Flowjo program, and bar graphs were created using Prism software. Assessment of statistical significance was performed by two-way ANOVA. Data are mean ± S.E.M of 20 subjects each. *p < 0.05, **p < 0.01, ***p < 0.001, ****p < 0.0001

Monocytes can be divided into three subsets based on their expression levels of CD14 and CD16 (FcγRIII): CD14^++^CD16^+^ are referred to as classical monocytes, CD14^+^CD16^+^ are referred to as intermediate monocytes, and CD14^+^CD16^++^ are referred to as non-classical monocytes. The increased level of CD16 in NMOSD and MS monocytes could be due to either an increase in intermediate or in non-classical subsets. This question could not be addressed using the CD14^+^ MACS-separation process, because the CD14^+^ beads were already occupied with CD14 molecules. Thus, to investigate the frequency of the three different monocyte subsets, peripheral blood mononuclear cells (PBMCs) from HC, MS, and NMOSD patients (*n* = 20 each) were stained for CD3, CD14, CD16, CD19, CD56, and CD66b to gate the monocyte subset (Additional file 1: Figure S2). The data shows that, in MS and NMOSD, the classical monocyte subset was decreased by 10% compared to that in HC monocytes (*p* < 0.001 for both). In addition, the frequencies of intermediate and non-classical subsets were increased by a percentage equivalent to the decrease in the classical monocyte frequency seen in MS and NMOSD monocytes, respectively (Fig. 4b). In fact, the frequency of non-classical monocytes in NMOSD was four-fold and two-fold higher than that in HC (*p* < 0.0001) and MS (*p* < 0.05), respectively. However, the frequency of intermediate monocytes in NMOSD was similar to, or lower than, that in HC (*p* = 0.4866) and MS (*p* < 0.05), respectively (Fig. 4a, b). Notably, a remarkable increase in the intermediate monocyte subset frequency was observed in MS (*p* < 0.05 for both), agreeing with previous reports [17, 24]. Overall, in NMOSD, the frequency of non-classical monocytes increased whereas the frequency of intermediate monocytes did not. This result implies that the constitutively increased level of CD16 observed previously in NMOSD (Fig. 3b) was mainly driven by an increase in non-classical monocytes.

To validate our data on the CD16 levels in HC, MS, and NMOSD, we employed a SPADE analysis. The data showed that NMOSD monocytes do indeed have increased CD16 expression and decreased CD14 expression level in the monocyte clusters compared to HC and MS (Fig. 4c).

### Non-classical monocytes in NMOSD exhibit constitutive IL-6 production

From our data, NMOSD monocytes exhibit increased inflammatory cytokine production, increased inflammatory surface molecules, and an increased frequency of the non-classical monocyte subset compared to HC monocytes (Figs. 3 and 4). Based on these results, it was still unclear which monocyte subset was responsible for the cytokine production and inflammatory characteristics. To address this, we purified monocytes using pan-monocyte isolation kit (Additional file 1: Figure S1-B) and stimulated monocytes with CD40L, identical to that used previously (Fig. 2) and compared cytokine production from each monocyte subset between three groups (*n* = 15 each).

**Fig. 5:**
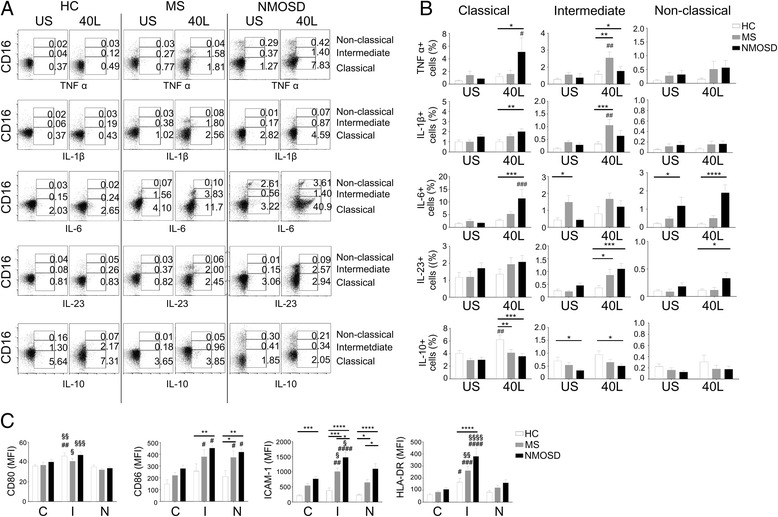
Intracellular cytokine staining of monocyte subsets in healthy control (HC), MS, and NMOSD patients. Monocytes were purified with MACS pan-monocyte isolation kit. Purified monocytes were treated with, or without, CD40L (1.0 μg/mL) for 6 h and then stained with the appropriate surface antigen and cytokine antibodies. Graphs are shown in representative data, where the *Y*-axis is CD16 expression, and the *X*-axis represents cytokine expression (TNFα, IL-1β, IL-6, IL-23, IL-10) (**a**). The bar graphs show the large-scale analysis of monocyte intracellular cytokine staining (*n* = 15) (**b**). Each cytokine is divided into two categories, a cultured condition (CD40L-stimulated or unstimulated) and monocyte subsets. Monocyte subsets were defined as classical, intermediate, and non-classical. The symbol “#” above the bar graphs indicates significance between control and CD40L for each group. (**c**) PBMCs were stained with various cell-type markers to analyze the monocyte subset population and surface molecules in each group (*n* = 10). The symbol “#” above the bar graphs indicates significance between the classical subsets, and the symbol “§” indicates significance between the non-classical subsets. Graphs were created using Flowjo and Prism software. Assessment of statistical significance was performed by two-way ANOVA. Data are mean ± S.E.M of 10 subjects each. *p < 0.05, **p < 0.01, ***p < 0.001, ****p < 0.0001. US unstimulated, I interferon-γ, 40 L CD40L

In the US condition, inflammatory cytokine-producing monocytes were rarely observed. Interestingly, however, a significant proportion of NMOSD non-classical monocytes constitutively produced IL-6 (*p* < 0.05) (Fig. 5a). In fact, the mean frequency of IL-6^+^ cells under US conditions, as well as following CD40L stimulation, was 60% of that of the total non-classical monocytes in NMOSD, which is three-fold higher than that of MS and HC monocytes (Additional file 1: Figure S3). In contrast, intermediate monocytes in MS showed an increased frequency of IL-6^+^ cells compared to HC monocytes in the US condition (Fig. 5a, b). Unlike IL-6, other cytokines (TNFα, IL-1β, IL-23, IL-10) were not constitutively increased in NMOSD, MS, and HC monocytes.

After CD40L stimulation, the frequency of the TNFα^+^ classical subset of NMOSD monocytes was increased five-fold relative to that of the same subset in HC monocytes (*p* < 0.05). There was also a modest increase in TNFα^+^ intermediate monocytes compared to HC monocytes (*p* < 0.05) (Fig. 5b). In the case of MS, previous reports have shown that the frequency of TNFα^+^ monocytes was similar to that of HC monocytes, but CD16^+^ purified MS monocytes produced a greater amount of TNFα than HC monocytes [17]. In our data, in MS monocytes, the frequency of the TNFα^+^ classical monocyte subset was similar to that of the same subset in HC monocytes, but the TNFα^+^ intermediate monocyte subset was upregulated by CD40L stimulation, being significantly different from the HC intermediate monocyte subset (*p* < 0.01) (Fig. 5b). This result suggests that the earlier observation on MS CD16^+^ monocytes could be largely influenced by intermediate monocytes. The IL-6^+^ classical monocyte subset of NMOSD monocytes were increased three- and five-fold compared to the IL-6^+^ classical monocyte subset of MS (*p* = n.s.) and HC monocytes (*p* < 0.001), respectively. In addition, the IL-6^+^ non-classical monocyte subset of NMOSD monocytes was increased two-fold relative to the same subset in HC monocytes (*p* < 0.0001). In this study, we found that IL-10 was mainly upregulated by CD40L stimulation in the classical monocyte subset of HC monocytes whereas other subsets were less affected (*p* < 0.01) (Fig. 5b). This observation has also been previously reported [27], which suggests that CD14^+^ purified monocytes produced greater amounts of IL-10 than CD16^+^ purified monocytes. Both NMOSD and MS monocytes showed impaired IL-10 production, since the frequencies of IL-10^+^ classical monocytes were decreased in both monocytes compared to HC monocytes (MS, *p* < 0.01; NMOSD, *p* < 0.001). In addition, the IL-10^+^ intermediate monocyte subset of NMOSD monocytes was significantly decreased compared to the same subset of HC monocytes (MS, *p* = n.s.; NMOSD, *p* < 0.05) (Fig. 5a, b).

We also analyzed and compared the expression pattern of cell-surface molecules (CD80, CD86, ICAM-1, HLA-DR) in these monocyte subsets (*n* = 10 each). The co-stimulatory molecules CD80 and CD86 showed a different pattern of expression in these monocyte subsets, with CD80 having a higher expression only in the intermediate monocyte subset than in the other subsets, but CD86 showed higher expression in both the intermediate and non-classical than in the classical subset (Fig. 5c). Interestingly, the ICAM-1 expression level in the intermediate monocyte subset was high in NMOSD and MS monocytes, but not in HC. For HLA-DR expression, intermediate monocyte subset expressed significantly higher than other subsets in all three groups, as previously reported [28, 29]. A comparison between disease groups showed that the NMOSD monocyte subsets had higher expression of these cell-surface molecules. In classical monocytes, the expression level of ICAM-1 was increased in NMOSD compared to that in HC classical monocytes (*p* < 0.001). In intermediate monocytes, the expression levels of CD86 (*p* < 0.01), ICAM-1 (*p* < 0.0001), and HLA-DR (*p* < 0.0001) in NMOSD were also increased compared to that in HC intermediate monocytes. In addition, ICAM-1 expression was the highest in NMOSD intermediate monocytes; MS intermediate monocytes also showed increased expression of ICAM-1 compared to HC intermediate monocytes (*p* < 0.001), but the expression was lower than in NMOSD intermediate monocytes (*p* < 0.05). In non-classical monocytes, the expression levels of CD86 (*p* < 0.001) and ICAM-1 (*p* < 0.0001) in NMOSD were increased compared to that in HC monocytes. Also, MS non-classical monocytes exhibited increased expression of CD86 (*p* < 0.05) and ICAM-1 (*p* < 0.05) compared to HC. However, ICAM-1 expression was higher in NMOSD non-classical monocytes than in MS non-classical monocytes (*p* < 0.05).

## Discussion

An increasing interest in the role of monocytes in the immune response has yielded findings that monocyte dysregulation is deeply involved in autoimmune diseases such as MS, SLE, and RA. For example, MS monocytes have been reported to be easily activated [30] and to have increased levels of inflammatory cytokine transcription and translation [31] and cell-surface molecule expression [32]. In this study, we hypothesized that NMOSD monocytes are dysregulated in much the same way that MS monocytes are. The data presented here demonstrate that NMOSD monocytes are readily activated and show increased production of inflammatory cytokines, decreased production of IL-10, increased expression of inflammatory surface molecules, and increased frequencies of a non-classical monocyte subset compared to HC monocytes. Interestingly, NMOSD monocytes have an even more inflammatory characteristic in some measures than MS monocytes, which are well-known to be highly inflammatory.

To examine monocyte cytokine production, we evaluated IL-6, TNFα, IL-1β, IL-23, and IL-10 levels. Among these cytokines, IL-6 is reported to play a critical role in the pathogenesis of NMOSD, and furthermore, a treatment targeting IL-6 has shown clinical benefit in NMOSD patients [33]. Not surprisingly, IL-6 production was found to be the highest in both unstimulated and CD40L-stimulated NMOSD monocytes compared to MS and HC monocytes (Figs. 1a and 5a). The presence of constitutive IL-6-producing non-classical monocytes could explain the reason why NMOSD monocytes produced such high levels of IL-6 under unstimulated condition. Along with IL-6, it has been reported that Th1- and Th17-related pro-inflammatory cytokines are over-expressed in the CSF of NMOSD patients [34]. TNFα is a Th1-related pro-inflammatory cytokine that is elevated in many autoimmune diseases, and its dysregulation characterizes many autoimmune diseases [35]. Likewise, IL-1β, which is associated with Th17 differentiation, is also involved in a number of autoimmune diseases, and neutralization of IL-1β in these autoimmune diseases can reduce disease severity [36]. In NMO-IgG seropositive rats, intra-striatal injection of IL-1β triggered the formation of NMO-like lesions [37]. In our study, the elevation of TNFα and IL-1β in NMOSD monocytes was observed under stimulated condition. IL-23, produced by myeloid cells, is one of the cytokines that is essential for Th17 differentiation [38], and Th17 is associated with NMOSD relapse [39]. We have found that IL-23 production was elevated in NMOSD. In line with the pro-inflammatory cytokines, IL-10, an anti-inflammatory cytokine, had a reciprocally imbalanced production. Previous reports on NMOSD showed that IL-10 level is decreased in the serum [40].

In MS, cell-surface molecules like co-stimulatory molecules are involved in disease exacerbation, are therapeutic targets [41], and can influence the age of disease onset [42]. Inhibition of these co-stimulatory molecules using CTLA-4-Fc in experimental autoimmune encephalomyelitis, an animal model of MS, has been effective in decreasing inflammation and demyelination [43], but there are few reports on NMOSD. CD80 and CD86 have been reported to be differentially expressed depending on the activation status. CD86 acts as an initial co-stimulatory ligand and is constitutively expressed, whereas CD80 is transiently expressed after activation [44, 45]. This was also observed in SLE, where CD86 was highly expressed in freshly isolated cells and CD80 was upregulated after culturing for 24 h in media [45]. In this study, CD86 expression was significantly higher in ex vivo CD16^+^ NMOSD and MS monocytes than in HC monocytes (Fig. 5c). In contrast, CD80 was not upregulated in ex vivo *monocytes* but showed a significant difference in expression upon stimulation (Fig. 3). These findings therefore agree with those of previous reports on the patterns of CD80 and CD86 expression. Other cell-surface molecules like ICAM-1 and HLA-DR have been shown to be involved in the initiation and propagation of autoimmune diseases [46, 47]. We found that the expression levels of ICAM-1 and HLA-DR were the highest in NMOSD monocytes both ex vivo (Fig. 5c) and upon stimulation (Fig. 3). These findings concerning the expression of cell-surface molecules also confirm the inflammatory characteristic of NMOSD monocytes.

Monocytes are divided into three subsets based on their expression of CD14 and CD16: CD14^++^CD16^+^ classical, CD14^+^CD16^+^ intermediate, and CD14^+^CD16^++^ non-classical monocytes. Although the individual roles and function of each subset are still being studied, it is assumed that these blood monocyte subsets represent stages in a developmental sequence, with non-classical monocytes being considered as the more mature monocytes [48]. Several groups have reported increased frequencies of circulating CD16^+^ monocytes in various autoimmune diseases [14, 15]. These CD16^+^ monocytes have been shown to actively shape T cell responses by favoring Th17 differentiation [49] and to facilitate T cell migration [17]. All these findings suggest that CD16^+^ monocytes are involved in the pathogenic processes of autoimmune diseases. Indeed, the frequency of the circulating non-classical monocyte subset was significantly higher in NMOSD than in HC and MS. More importantly, these non-classical NMOSD monocytes constitutively produce IL-6, but this was not the case for non-classical MS and HC monocytes. Upon stimulation with CD40L, NMOSD monocytes produced higher amounts of IL-23 and IL-6 compared to MS and HC monocytes. Taken together, these findings indicate a crucial role for non-classical monocytes in the generation and maintenance of a pathologically relevant Th17 environment in NMOSD patients.

In this study, we used CD40L and/or IFN-γ to mimic encountering with activated T cells. When each of these stimuli was used by themselves, the effects on inflammatory cytokine production and cell-surface molecule upregulation were robust. However, co-treatment of these two stimuli did not produce synergetic effects. One possible explanation of this is that both ligands signal through Jak-STAT pathway components [50], which would preclude any additive effect, but a further analysis is warranted.

Understanding the adaptive immune system in NMOSD is important to target molecules of immune attack. A series of observation suggest a proinflammatory humoral response in NMOSD [51]. AQP4-IgG, which is detected in approximately 75~80% of NMOSD patients [52], has been shown to reproduce the cardinal features of disease pathology [53–56], supporting the direct role of this autoantibody in disease development. Nevertheless, AQP4-IgG alone is not sufficient to provoke the disease. Most likely, AQP4-specific T cells are required in the peripheral immune compartment to help generate the class-switched autoantibodies from B cells, as well as to the development of NMOSD lesions in the CNS. In particular, T helper 17 cells, which can provide B cell help and induce tissue inflammation, may also play a key role in the pathogenesis of NMOSD [57].

This study has several limitations. First, none of the enrolled NMOSD patients were treatment-naïve. Therefore, the effects of disease-modifying therapies cannot be excluded, although the effect of high-dose steroids was excluded. Nevertheless, it is intriguing that NMOSD monocytes showed a stronger inflammatory phenotype than MS monocytes in some measures despite the use of immunosuppressants (rituximab, RTX; mycophenolate mofetil, MMF; prednisolone, PD; azathioprine, AZT) in NMOSD patients and immunomodulators (glatiramer acetate, interferon-β) in a majority of MS patients. Second, the disease durations for MS (60.4 ± 42.45) and NMOSD (102.1 ± 84.10) were not matched. It is possible that this could affect the data and the monocyte subset frequencies in treatment-naïve or NMOSD patients in an early stage of disease could be different from what we have observed. Third, changes in monocyte dysregulation at relapse have not been evaluated. Lastly, other functions of NMOSD monocytes, like phagocytosis or migration, have not been analyzed. Altogether, these warrant further studies.

## Conclusions

In conclusion, we demonstrated a perturbation of monocyte activation in NMOSD patients. Increased expression of cell-surface molecules and a reciprocal dysregulation of inflammatory and anti-inflammatory cytokines in NMOSD monocytes show clear evidence of an altered monocyte inflammatory response. Further analysis showed that the CD14^+^CD16^++^ non-classical monocyte subset was more abundant in NMOSD monocytes than in HC or MS monocytes. Closer observation of cytokine production revealed NMOSD non-classical monocyte subset has dysregulated IL-6 production, a phenotype which has been reported to be highly associated with NMOSD pathogenesis.

## Additional file

Additional file 1: Figure S1. Gating strategy for both CD14+ monocyte purification and pan-monocyte purification. Cell viability was checked by using PI staining. Monocytes were stained with CD3, CD14, CD19, CD56 and CD66b antibody for both before and after purification samples. **Figure S2.** Identification of peripheral blood monocyte subsets by flow cytometry. Monocyte subsets were identified by negative selection. Neutrophils, NK cells, B and T cells were excluded by using conventional bivariate scatterplots of side scatter signal versus cell-specific markers. The remaining population was selected with HLA-DR, and was then sub-classified into three monocyte subsets using CD14 versus CD16. Graphs were created using Flowjo software. **Figure S3.** Percentage of IL-6 positive cells in non-classical monocytes from healthy controls (HC), MS, and NMOSD patients (*n* = 15). The percentage of IL-6 positive cells in the non-classical monocyte population was calculated for HC, MS, and NMOSD. Graphs were created using Flowjo software. Assessment of statistical significance was performed by two-way ANOVA followed by Dunnett’s multiple comparisons test. **P* < 0.05, ***P* < 0.01, ****P* < 0.001, *****P* < 0.0001.

## Abbreviations

AQP4: Aquaporin 4 water channel protein
CNS: Central nervous system
CSF: Cerebrospinal fluid
HC: Healthy control
MS: Multiple sclerosis
NMOSD: Neuromyelitis optica spectrum disorder
US: Unstimulated
