# Supplementary material for: Increased frequency of IL-6-producing non-classical monocytes in neuromyelitis optica spectrum disorder
Source: J Neuroinflammation. 2017 Sep 25;14:191. doi: 10.1186/s12974-017-0961-z (PMC5613387; doi:10.1186/s12974-017-0961-z)
Supplement: Additional file 1: Figure S1. — Gating strategy for both CD14+ monocyte purification and pan-monocyte purification. Cell viability was checked by using PI staining. Monocytes were stained with CD3, CD14, CD19, CD56 and CD66b antibody for both before and after purification samples. Figure S2. Identification of peripheral blood monocyte subsets by flow cytometry. Monocyte subsets were identified by negative selection. Neutrophils, NK cells, B and T cells were excluded by using conventional bivariate scatterplots of side scatter signal versus cell-specific markers. The remaining population was selected with HLA-DR, and was then sub-classified into three monocyte subsets using CD14 versus CD16. Graphs were created using Flowjo software. Figure S3. Percentage of IL-6 positive cells in non-classical monocytes from healthy controls (HC), MS, and NMOSD patients (n = 15). The percentage of IL-6 positive cells in the non-classical monocyte population was calculated for HC, MS, and NMOSD. Graphs were created using Flowjo software. Assessment of statistical significance was performed by two-way ANOVA followed by Dunnett’s multiple comparisons test. *P < 0.05, **P < 0.01, ***P < 0.001, ****P < 0.0001. [file 12974_2017_961_MOESM1_ESM.pptx]

## Slide 1
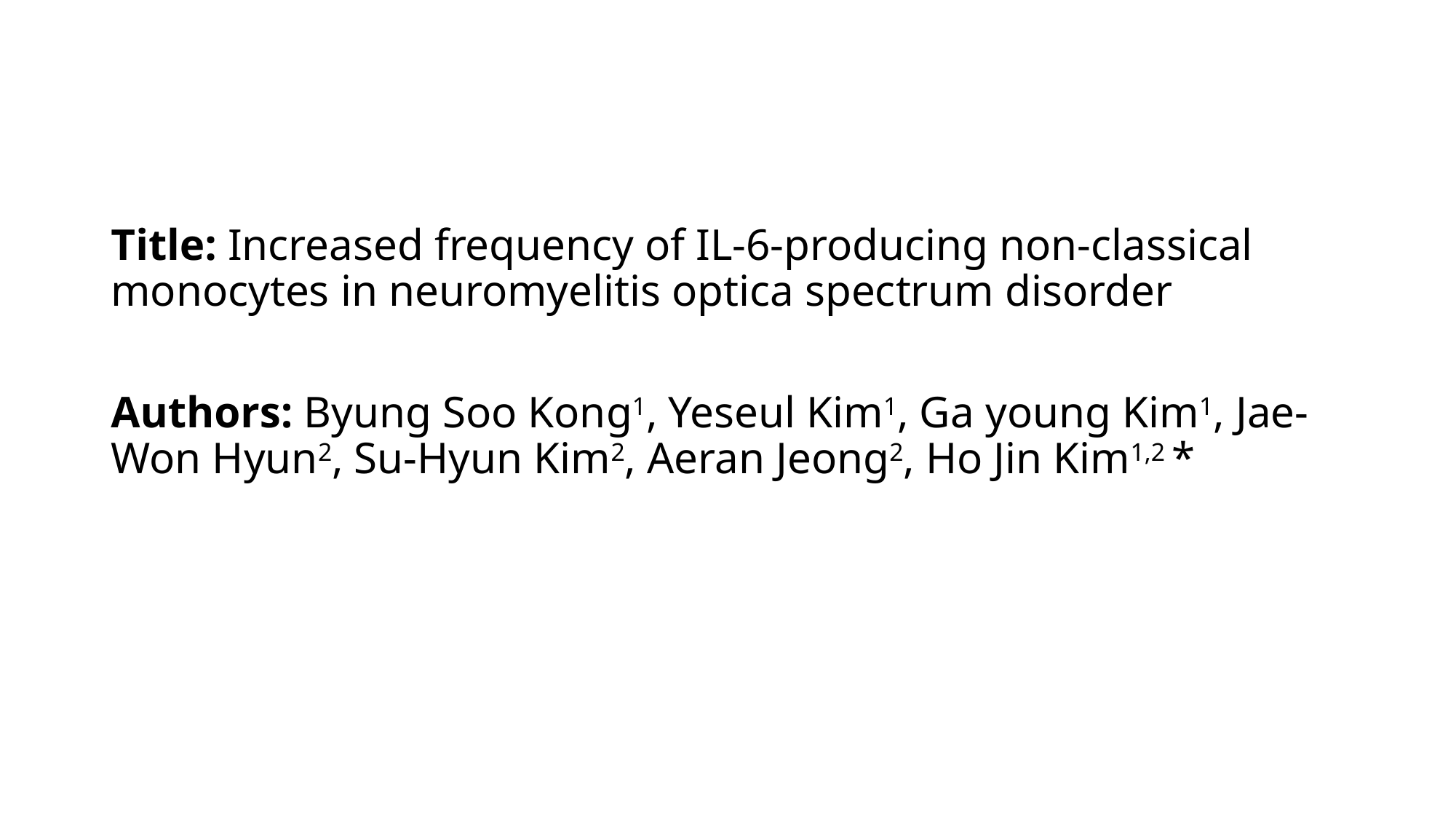

Title: Increased frequency of IL-6-producing non-classical monocytes in neuromyelitis optica spectrum disorder
Authors: Byung Soo Kong1, Yeseul Kim1, Ga young Kim1, Jae-Won Hyun2, Su-Hyun Kim2, Aeran Jeong2, Ho Jin Kim1,2 *

## Slide 2
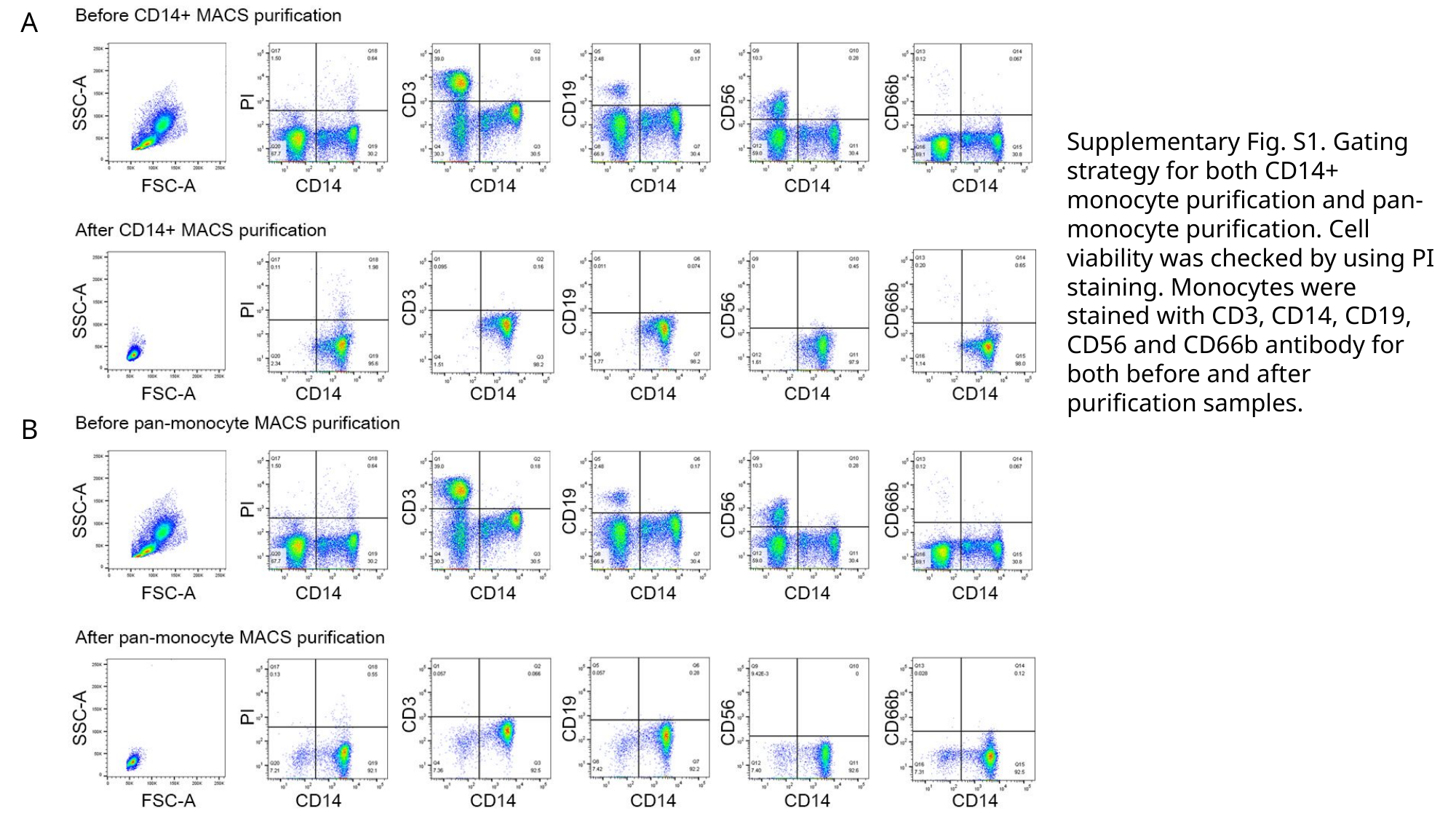

A
Supplementary Fig. S1. Gating strategy for both CD14+ monocyte purification and pan-monocyte purification. Cell viability was checked by using PI staining. Monocytes were stained with CD3, CD14, CD19, CD56 and CD66b antibody for both before and after purification samples.
B

## Slide 3
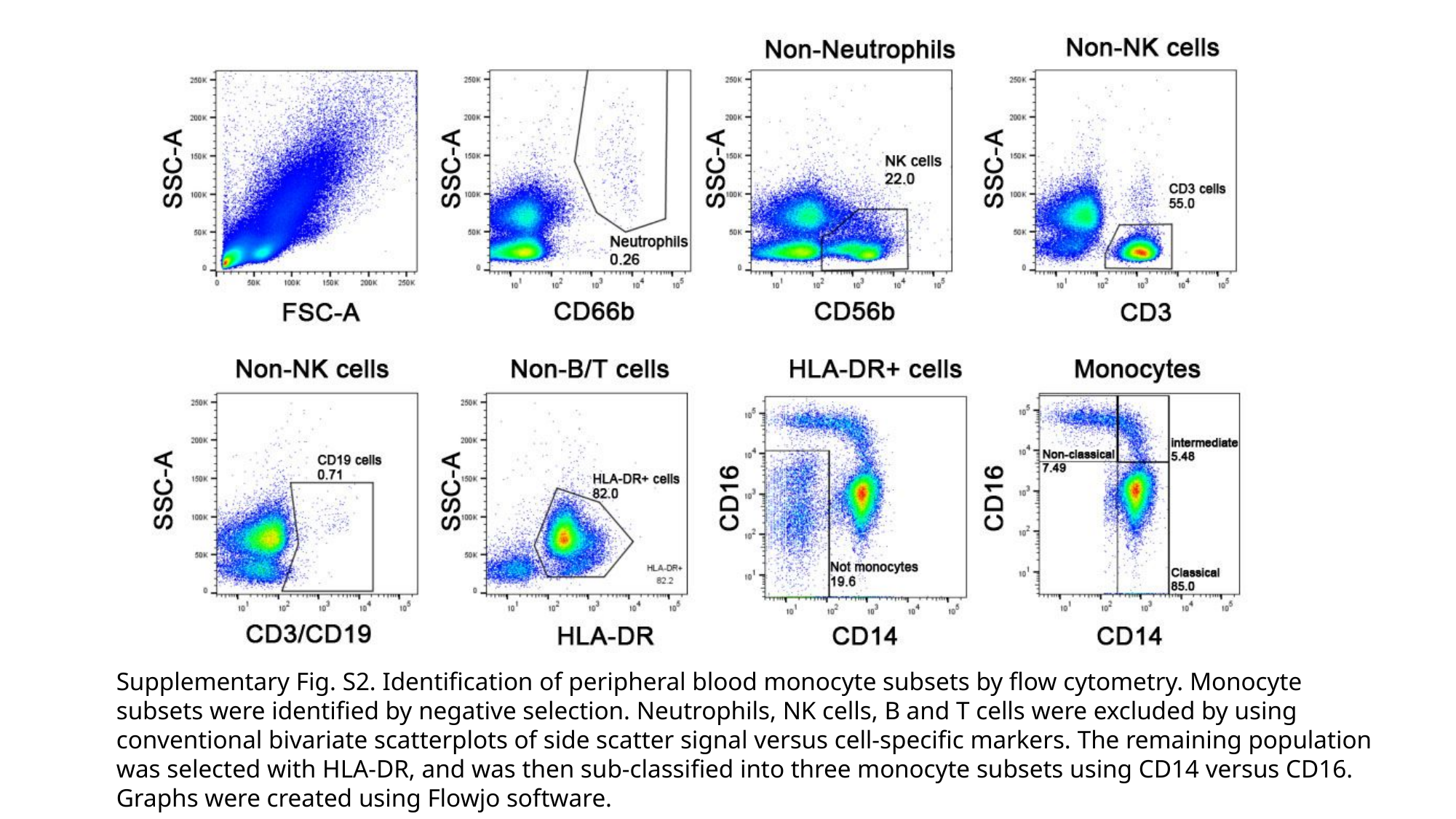

Supplementary Fig. S2. Identification of peripheral blood monocyte subsets by flow cytometry. Monocyte subsets were identified by negative selection. Neutrophils, NK cells, B and T cells were excluded by using conventional bivariate scatterplots of side scatter signal versus cell-specific markers. The remaining population was selected with HLA-DR, and was then sub-classified into three monocyte subsets using CD14 versus CD16. Graphs were created using Flowjo software.

## Slide 4
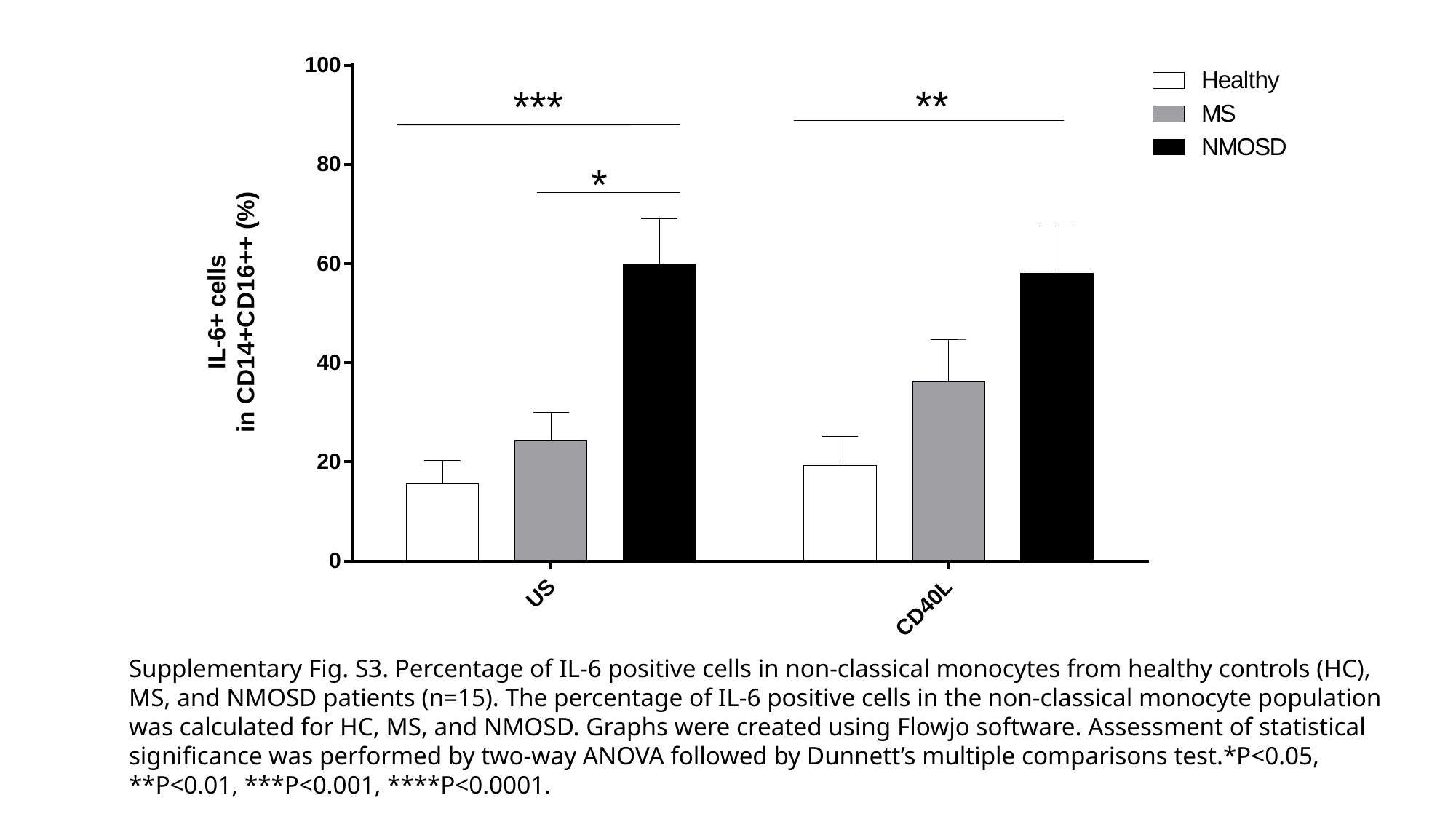

**
***
*
Supplementary Fig. S3. Percentage of IL-6 positive cells in non-classical monocytes from healthy controls (HC), MS, and NMOSD patients (n=15). The percentage of IL-6 positive cells in the non-classical monocyte population was calculated for HC, MS, and NMOSD. Graphs were created using Flowjo software. Assessment of statistical significance was performed by two-way ANOVA followed by Dunnett’s multiple comparisons test.*P<0.05, **P<0.01, ***P<0.001, ****P<0.0001.
